# Multifunctional PDO Thread Coated with Mg(OH)_2_/ZnO Nanoparticles and Asiaticoside for Improved Facial Lifting

**DOI:** 10.3390/pharmaceutics15092220

**Published:** 2023-08-28

**Authors:** Dong Min Kim, Seung-Woon Baek, Jeong Min Park, Da-Seul Kim, Semi Lee, Jun-Kyu Lee, Chun Gwon Park, Dong Keun Han

**Affiliations:** 1 Department of Biomedical Science, CHA University, 335 Pangyo-ro, Bundang-gu, Seongnam-si 13488, Republic of Korea; kimdmnice@gmail.com (D.M.K.); baiksw830@g.skku.edu (S.-W.B.); operationx1414@gmail.com (J.M.P.); dptmf4011@cau.ac.kr (D.-S.K.); a41915343@gmail.com (S.L.); jklee2020@chauniv.ac.kr (J.-K.L.); 2 Department of Biomedical Engineering, SKKU Institute for Convergence, Sungkyunkwan University (SKKU), 2066 Seobu-ro, Jangan-gu, Suwon-si 16419, Republic of Korea; chunpark@skku.edu; 3 Department of Intelligent Precision Healthcare Convergence, SKKU Institute for Convergence, Sungkyunkwan University (SKKU), 2066 Seobu-ro, Jangan-gu, Suwon-si 16419, Republic of Korea

**Keywords:** facial rejuvenation, lifting thread, polydioxanone, magnesium hydroxide, zinc oxide, asiaticoside

## Abstract

As interest in skin aesthetics increases, treatments to suppress aging are increasing. Among them, a facelift is the most effective procedure for improving wrinkles. However, side effects including inflammatory reactions occur due to the limitations of the PDO thread itself used during the procedure. In this paper, to improve the function of PDO thread, inorganic particles such as magnesium hydroxide (MH) and zinc oxide (ZO) and a biologically active agent, asiaticoside, were coated on the surface of PDO thread using ultrasonic coating technology. The coated thread exhibited excellent biocompatibility, promoted collagen synthesis, reduced inflammation, and stimulated angiogenesis in vitro and in vivo. The multifunctional PDO thread has shown promising potential for skin regeneration without inducing fibrosis. Such a practical coating system and the developed multifunctional PDO thread suggest new possibilities for developing safer and more effective materials in cosmetic and regenerative medicine to prevent aging and improve skin aesthetics.

## 1. Introduction

As aging increases, the skin aesthetics and aging procedures are increasing. Aging progresses via various mechanisms such as collagen loss and muscle degeneration [1]. To suppress aging, treatments such as Botox, fillers, and facelifts are being performed to improve wrinkles, which are a prominent sign. Botox is a procedure that temporarily inhibits facial muscle contraction to diminish wrinkles, but it carries the risk of facial nerve paralysis as a potential side effect. Dermal fillers, on the other hand, involve restoring skin volume to reduce wrinkles but may lead to side effects like blurred vision and numbness around the eyes [2]. However, if the skin is severely saggy, wrinkle improvement using Botox and fillers is limited, and a facelift is inevitable [3]. The facelift improves wrinkles by inserting a lifting thread under the skin and is preferred due to its relatively small incision and short procedure time compared to cosmetic surgery [4]. The lifting threads used for a facelift are manufactured using various biodegradable polyesters such as poly(L-lactic acid) (PLLA), poly(ε- caprolactone) (PCL), and poly(dioxanone) (PDO), and the recently developed PDO threads are recognized for their excellence in the biodegradation period and biocompatibility [5]. It is well known that PDO is non-toxic to the human body because it is absorbed in the body and decomposed into CO_2_ and H_2_O and is generally decomposed within approximately 6 months [6]. However, polyester including PDO produces acidic by-products in the process of hydrolysis in the human body. The acid by-products acidify the surrounding tissue, causing non-infectious inflammation [7]. Inflammation within the skin can accelerate aging and induce fibrosis [8].

To improve the non-infectious inflammatory response by acidic by-products of aliphatic polyester, the pH-neutralizing effect of magnesium hydroxide (MH) was utilized in our previous studies [9,10,11]. MH, a basic inorganic particle, exposed to an acidic environment reacts with hydrogen ions and dissociates into magnesium and hydroxide ions, resulting in a pH-neutralization effect. In addition, magnesium ions released by MH maintain the homeostasis of cells from various stimuli [7].

Zinc oxide (ZO), as a safe and nontoxic material recognized by the FDA, has recently received much attention in biotechnology, medicine, biology, and physics [12,13,14,15]. Recently, ZO has been reported by many researchers as a continuous nitric oxide (NO) release material by the oxidation-reduction reaction with NO donor [16,17]. NO serves as a signaling molecule responsible for essential biological functions in all tissues of the human body, such as the cardiovascular, nervous, and immune systems [18,19,20]. In particular, NO in the skin induces wound healing by being involved in an inflammatory response, immune response, angiogenesis, and collagen formation [21,22].

Asiaticoside (ATS) extracted from *Centella asiatica* has a triterpene structure and involves wound healing with anti-inflammation and collagen formation [23]. ATS induces the synthesis of type I collagen via the TGF-β receptor I kinase-independent Smad activation pathway [24]. In addition, research on angiogenesis by ATS has recently been reported [25].

In this study, we devised an ultrasonic coating technology of functional inorganic particles that can reduce the occurrence of biological side effects. Moreover, a sustained release system of ATS that induces wound healing and skin regeneration was suggested. We demonstrated collagen synthesis, an anti-inflammatory effect, and angiogenesis by the coated multifunctional PDO threads in vitro and in vivo. This practical coating system and the developed multifunctional PDO thread suggest new possibilities for developing safer and more effective materials in cosmetic and regenerative medicine for anti-aging and improving skin aesthetics.

## 2. Materials and Methods

### 2.1. Materials

Poly(dioxanone) (PDO) lifting thread (Secret Line) was provided by Hyundai Meditech (Gangwon, Republic of Korea). Poly(DL-lactide) (PDLLA, R205S) was acquired from Evonik Industries AG (Essen, Germany). Poly(L-lactide) (PLLA, Purasorb PL 32) was purchased from Corbion (Amsterdam, The Netherlands). Magnesium hydroxide (MH), zinc oxide (ZO), α-lipoic acid (ALA), L-glutathione reduced (GSH), and S-nitroso-N-acetyl-DL-penicillamine (SNAP) were purchased from Sigma-Aldrich (St. Louis, MO, USA). Oleic acid was purchased from TCI (Tokyo, Japan). Asiaticoside (ATS) was purchased from MedchemExpress (Monmouth junction, NJ, USA). Chloroform and Tetrahydrofuran (THF) were obtained from Daejung Co., Ltd. (Seoul, Republic of Korea). A 4-amino-5-methylamine-2,7-difluorofluorescein diacetate (DAF-FM DA) was provided by Cayman Chemical (Ann Arbor, MI, USA). Human dermal fibroblasts (hDFs) were purchased from ATCC (Manassas, VA, USA). Human umbilical vein endothelial cells (HUVECs) and an EGM-2 media bullet kit were purchased by Lonza (Basel, Switzerland). Dulbecco’s Modified Eagle Medium (DMEM), fetal bovine serum (FBS), and a phosphate-buffered saline (PBS) solution were purchased by Hyclone (Cytiva, MA, USA). The antibiotic antimycotic solution (A/A) and nuclease-free water were obtained from Gibco (Thermo Fisher Scientific, Waltham, MA, USA). A cell-count kit (CCK-8) was obtained from Dongin LS (Hwaseong, Republic of Korea). The Calcein AM/EthD-1 staining kit and nuclease-free water were obtained from Invitrogen (Thermo Fisher Scientific, Waltham, MA, USA). The TAKARA RT reagent kit and PrimeScript RT Reagent Kit (Perfect Real Time) were provided by Takara Bio Inc. (Tokyo, Japan). IL-6 and IL-8 enzyme-linked immunosorbent assay (ELISA) kits were purchased from R&D Systems (Minneapolis, MN, USA). The HRP/DAB (ABC) detection IHC kit was obtained from Abcam (Cambridge, UK).

### 2.2. Synthesis and Characterization of Surface-Modified Inorganic Particles

To improve dispersion stability in a hydrophobic coating solvent, MH was surface-modified using oleic acid (OA) to obtain MO. For this, 0.75 g oleic acid was dissolved in 70 mL of hexane and added to 3 g MH dispersed in distilled water. The mixture was then reacted at 60 °C for 4 h with stirring. Similarly, ZA nanoparticles were synthesized to enhance dispersion stability in the hydrophobic coating solvent and facilitate NO generation, as described in our previous study [16]. This was achieved through conjugation between ZO and α-lipoic acid (ALA). For the synthesis, 1.5 g α-lipoic acid was dissolved in 50 mL of chloroform and added to 1.5 g ZO. The mixture was then reacted at 60 °C for 24 h with stirring under a vacuum. To remove unreacted substances from MO and ZA, the products were washed using chloroform and centrifuged at 10,000 rpm three times, respectively.

To analyze the chemical bond of the surface-modified inorganic particles, attenuated total reflection-Fourier transform infrared (ATR-FTIR; PerkinElmer, Waltham, MA, USA) analysis was performed with a spectral resolution of 32 scans and 4 cm^−1^ in the 400–4000 cm^−1^ range. The binding ratio between the inorganic particles and the materials used for surface modification was measured using thermogravimetric analysis (TGA; TGA-4000, PerkinElmer, Waltham, MA, USA), with a heating range of 30 to 800 °C at a rate of 10 °C/min under a nitrogen atmosphere. The size distribution of the MO and ZA was measured in chloroform using dynamic laser scattering (DLS; Malvern Panalytical, Malvern, UK).

### 2.3. Preparation and Characterization of Multifunctional PDO Threads

The multifunctional PDO thread was fabricated through a two-step coating process. The first coating solution was prepared by suspending MO (6 mg/mL) and ZA (3 mg/mL) in chloroform, with the addition of dissolved PLLA (0.3 wt%). Another coating solution was created by dispersing MO (20 phr) and ZA (10 phr) in a PDLLA solution (0.3 wt% in chloroform), which also contained ATS (3 phr in THF). The surface of the multifunctional PDO thread (P) was then sequentially coated with these prepared solutions using an ultrasonic spray coater (Noanix, Cheongju, Republic of Korea) to obtain MO-coated PDO (PM), ZA-coated PDO (PZ), and ATS-coated PMZ (PMZA), respectively.

The dispersion stability of MO and ZA in chloroform was investigated by measuring absorbance at 550 nm using a microplate reader (SpectraMax M2 Microplate Reader, Molecular Devices, San Jose, CA, USA) [26]. The coated surfaces and distribution of MO and ZA on PDO threads were visualized using field emission-scanning electron microscopy (FE–SEM; S–4800, Hitachi, Tokyo, Japan) equipped with energy-dispersive spectroscopy (EDS). The amount of MO and ZA was investigated by inductively coupled plasma–optical emission spectroscopy (ICP-OES, Optima 8000, PerkinElmer, Waltham, MA, USA). The released ATS was measured using high-performance liquid chromatography (HPLC; Thermo Scientific Inc., Waltham, MA, USA). The separation was performed using a Luna 100u c150(460) 3A, LC column (Phenomenex, Torrance, CA, USA) with 3-micron particle size and 18 × 2 mm dimensions. The mobile phase consisted of 70% acetonitrile, 25% methanol, and 5% water, with a 1 mL/min flow rate. A total of 10 μL of each sample was injected into the column for analysis. The detection wavelength was set at 265 nm, and the study was performed for 15 min. The NO generated by the multifunctional PDO threads was investigated using a DAF-FM DA fluorescent probe. The PDO threads were immersed in PBS solution with 50 μM DAF-FM DA, 50 μM GSH, and 50 μM SNAP at 37 °C for 12 h Then, the recovered supernatant was visualized as a fluorescence-labeled organism bioimaging instrument (FOBI; CellgenTEK Co., Cheongju, Republic of Korea), and quantitative fluorescence intensity was measured as a fluorescence microplate reader (SpectraMax M2 Microplate Reader, Molecular Devices, San Jose, CA, USA).

### 2.4. Biocompatibility and Collagen Deposition Ability

The hDFs were cultured at 37 °C, 5% CO_2_ in high-glucose DMEM containing 1% antibiotic antimycotic and 10% FBS. The hDFs were seeded in a 24-well culture plate at a density of 1 × 10^4^ cells/well. The multifunctional PDO threads (40 mm) were treated on 24-well inserts in each well for 24 h. The live/dead fluorescence images and cell viability were measured using the Calcein AM/EthD-1 staining kit and the CCK-8 assay according to the provided instructions, respectively.

The hDFs were seeded at a density of 3 × 10^4^ cells on 24-well plates and the PDO threads were processed and incubated for 24 h. After processing, the cells were rinsed with PBS solution and fixed with 4% PFA solution for 15 min, permeabilized with 0.3% triton for 30 min, and finally blocked by 1% BSA solution for 30 min. Cells were probed with a COL1 antibody (Santa Cruz Biotechnology; Santa Cruz, CA, USA) in 5% BSA/0.1% Triton X-100 solution at 4 °C overnight. After rinsing with PBS solution and treated secondary antibody (Alexa-Fluor 568 goat anti-rabbit; Molecular Probes Inc., Eugene, OR, USA) in 5% BSA/0.1% Triton X-100 for 1 h at room temperature. Following this, they were rinsed 3 times with the PBS solution and mounted using Vectashield mounting medium/DAPI (Vector, CA, USA). The cells were observed using fluorescent microscopy.

### 2.5. RNA Extraction and Quantitative Real-Time PCR

The hDFs were seeded in 6-well culture plates at a density of 1 × 10^5^ cells/well, and after 24 h, a 40 mm multifunctional PDO thread was treated with an insert. The RNA of cells was then extracted after 24 h. The RNA from cells was extracted using the AccuPrep RNA Extraction Kit. The extracted RNA was reverse-transcribed into cDNA using the PrimeScript RT Reagent Kit. Quantitative real-time PCR was performed using Power SYBR™ Green PCR Master Mix. All values were calculated using 18s rRNA and the 2^−ΔΔCt^ method.

Primer sequences (forward/reverse) were designed as follows: 18S (5′-GCAATTATTCCCCATGAACG-3′/5′-GGGACTTAATCAACGCAAGC-3′), Transforming growth factor-β1 (TGF-β1; 5′-GACTTTTCCCCAGACCTCGG-3′/5′-ATAGGGGATCTGTGGCAGGT-3′), collagen type 1 α1 (COL1A1; 5′-CCCAAGGCTTCCAAGGTC-3′/5′-CCCAAGGCTTCCAAGGTC-3′), tumor necrosis factor α (TNF-α; 5′-AGCCCATGTTGTAGCAAACC-3′/5′-TCTCAGCTCCACGCCATT-3), interleukin 1 β (IL-1β; 5′- TACCTGTCCTGCGTTGAA -3′/5′- TCTTTGGGTAATTTTTGGGATCT -3), interleukin-6 (IL-6; 5′-GATGGAGTACAAAAGTCCTGATCCA-3′/5′-CTGCAGCCATGGTTCTGT-3′), hypoxia-inducible factor-1α (HIF-1 α; 5′-TTTTTCAAGCAGTAGGAATT-3′/5′-GTGATGTAGTAGCTGCATGA-3), hepatocyte growth factor (HGF; 5′-CAGCATGTCCTCCTGCATC-3′/5′-TCTTTTCCTTTGTCCCTCTGC-3′), and vascular endothelial growth factor (VEGF; 5′-ACTGGACCCTGGCTTTACTG-3′/5′-TCTGCTCCCTTCTGTCGT-3′).

### 2.6. Quantitative Analysis of Inflammatory Cytokines

The hDFs were seeded in 24-well culture plates at a density of 1 × 10^4^ cells/well, and after 24 h, the degradation products of multifunctional PDO threads were treated. The degradation products of multifunctional PDO threads were prepared by immersing PBS solution at 60 °C for 21 days. Quantitative ELISA analysis was performed using media harvested from hDFs following the manufacturer’s instructions.

### 2.7. Angiogenic Capability

In order to assess the effect of wound closure of the PDO threads, the scratch method on cell monolayers was utilized. HUVECs (1.5 × 10^5^ cells/well) were seeded into a 6-well plate and incubated for 1 day. Cells grown as a continuous layer were scratched using a 1 mL pipette tip and washed with PBS solution. The PDO threads were co-cultured with the inserts and the area of the wound was observed after 12 h using optical microscopy (CKX53, Olympus, Tokyo, Japan). Healing of the wound area was measured using Image J software (National Institutes of Health, Bethesda, MD, USA). To evaluate the effect of angiogenesis of the PDO threads, 300 uL of the Matrigel matrix (356234, Corning, Steuben County, NY, USA) was applied to the 24-well plates and incubated at 37 °C for 1 h. HUVECs (1.2 × 10^5^ cells/well) were seeded and the PDO threads were co-cultured with Transwell inserts. After 16 h, the inserts were removed, and the cells were stained with calcein AM (4 µmol) following the instructions of the manufacturer. The cells stained with calcein AM were observed using fluorescence microscopy. The branch point and tube length of each group were quantified using Image J software.

### 2.8. Animal Test and Histological Evaluation

All animal procedures were performed in accordance with the Laboratory Animal Management and Use Guidelines of CHA University and approved by the Institutional Animal Care and Use Committee (IACUC220184). Male BALB/c mice aged 6 weeks (Orient Bio Inc., Seongnam, Republic of Korea) were used in the study. The mice were anesthetized with isoflurane (Terrell Isoflurane, Piramal Critical Care Inc., Bethlehem, PA, USA). A 5 mm skin incision was made and transplanted into the left and right subcutaneous regions of 5 mice in each group. After 6 weeks post-transplantation, the animals were sacrificed and evaluations were conducted. Tissue samples were embedded in paraffin and cut into 5 μm sections. The tissue sections were stained with Hematoxylin and Eosin (H&E; Abcam, Cambridge, UK) and Masson’s Trichrome (MT; Vitro Vivo Biotech, Rockville, MD, USA) according to the manufacturer’s instructions. mRNA from tissues of mice was isolated using TRIzol reagent (Thermo Scientific Inc., Waltham, MA, USA). The extracted RNA was reverse-transcribed into cDNA. Quantitative real-time PCR was performed using Power SYBR™ Green PCR Master Mix. All values were calculated using 18s rRNA and the 2^−ΔΔCt^ method. Primer sequences (forward/reverse) were designed as follows: COL1A1 (5′-TTCAGGTCCAATGGGTCCC-3′/5′-AGGCTCTCCCTTAGGACCAG-3′) and IL-6 (5′-ACGGCCTTCCCTACTTCACA-3′/5′-CATTTCCACGATTTCCCAGA-3’). Immunohistochemistry was performed with anti-CD31 (ab28364, Abcam 1:500) as primary antibodies (4 °C overnight) and an ABC detection IHC kit.

### 2.9. Statistical Analysis

All experiments were repeated at least three times. The results are presented as means ± SD. The statistical significance of observed differences was evaluated by a one-way analysis of variance using Tukey’s method and GraphPad Prism 9.0 software (GraphPad Software Inc., San Diego, CA, USA). * *p* < 0.05, ** *p* < 0.01, *** *p* < 0.001, and ^#^ *p* < 0.0001 indicate statistically significant differences.

## 3. Results and Discussion

### 3.1. Characterization of Multifunctional PDO Threads

**Figure 1:**
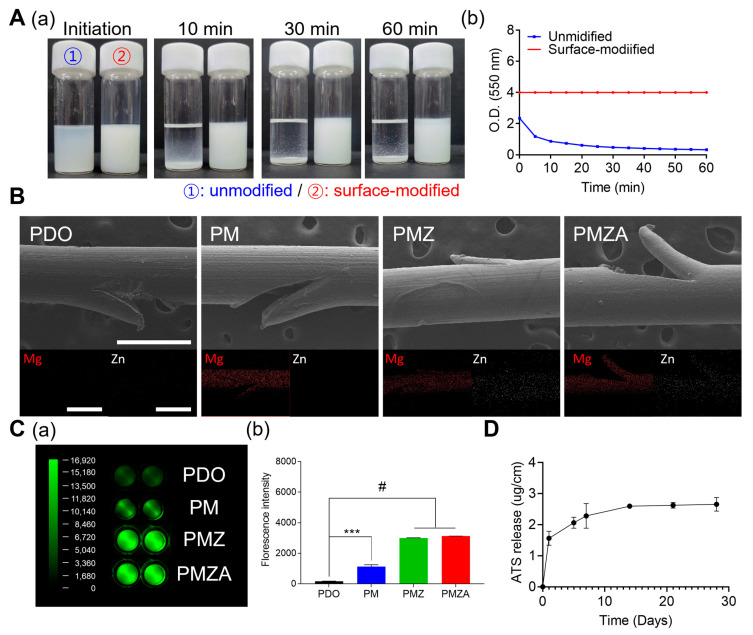
Characterization of the multifunctional PDO threads. (**A**) Dispersion images (a) and turbidity assay (b) for the dispersion evaluation. (**B**) Representative SEM and EDS mapping (Mg and Zn) images. (**C**) NO generation ability in the presence of 50 μM GSH and 50 μM SNAP; fluorescence images (a) and quantitative fluorescence intensity (b). (**D**) ATS release behavior for 28 days. *** *p* < 0.001, and ^#^ *p* < 0.0001 indicate statistically significant differences, respectively.

MH was chosen for its ability to neutralize acidic by-products of polyester. However, it faced challenges in achieving excellent dispersion stability within the polyester matrix due to its inherent hydrophilic properties. To overcome this issue, surface modification of MH was performed using oleic acid. The binding between MH and oleic acid was evidenced through ATR-FTIR spectra by the presence of an -OH stretching peak at 3698 cm^−1^ from MH and a C=O stretching peak at 2920–2850 cm^−1^ from oleic acid in the MO (Figure S1A). Additionally, the amount of oleic acid bound to the surface of MH was quantified to be 4.71% through TGA analysis (Figure S1B). MH was observed to aggregate and precipitate in organic solvents, making it challenging to accurately measure its size using DLS. In contrast, the MO exhibited high dispersion stability and maintained a nano size of 255.2 nm (Figure S1C). As reported in our previous study, ZA had improved NO-generating ability and dispersion stability in hydrophobic materials compared to ZO. As previously analyzed, the synthesis of ZA was demonstrated using ATR-FTIR, TGA, and DLS (Figure S2). The FTIR spectrum of ZA included both the -OH of ZO and the C=O of α-lipoic acid peaks, and it was found that α-lipoic acid was bound by 9.8% through TGA analysis. The size of ZA was 109 nm. In order to deliver the successfully synthesized two surface-modified inorganic particles and ATS as above to the PDO thread, a coating solution containing the biodegradable polymer was prepared and the dispersion stability was evaluated. Compared to the immediate precipitation of unmodified inorganic particles in the coating solution, high dispersion stability was secured in the coating solution including surface-modified inorganic particles for 1 h (Figure 1A(a)). Since fine precipitates can also affect coating uniformity, turbidity analysis was performed and the dispersion stability of coating solutions containing surface-modified inorganic particles was demonstrated to be excellent (Figure 1A(b)). The multifunctional PDO threads were coated using an ultrasonic coater. The coated surface and distribution of MO and ZA were displayed using FE-SEM and EDS mapping in Figure 1B. Each group did not display a significant change in the surface even after coating, and cog was also maintained. In addition, Mg and Zn elements used as indicators of MO and ZA, respectively, were evenly distributed over the entire area of the PDO threads. Since PDO threads require strong tensile force during a facelift, the effect on mechanical properties during the coating process was investigated (Figure S3). As a result, tensile strength, elongation, and Young’s modulus of multifunctional PDO threads maintained the same strength as before coating. Table 1 shows the loading amount. MH, ZO, and ATS were coated on the multifunctional PDO threads at approximately 22, 13, and 10 mg, respectively. The NO generation by multifunctional PDO threads was treated with 50 μM of SNAP and GSH and visualized and quantified through DAF-FM (Figure 1C). The PDO threads did not generate NO. On the other hand, the NO-generating by PMZA was higher than other groups by catalysis of MO and ZA. Both MH and ZO have a NO-generating ability, but ZO has the most excellent NO-generating capacity among numerous inorganic particles [17]. Moreover, the ability to generate NO of ZA was improved by α-lipoic acid [16]. The released ATS by PMZA was analyzed for 28 days, as shown in Figure 1D. ATS was rapidly released over 5 days and then slowly, with approximately 30% released over 28 days.

### 3.2. Biocompatibility and Collagen Deposition Ability

**Figure 2:**
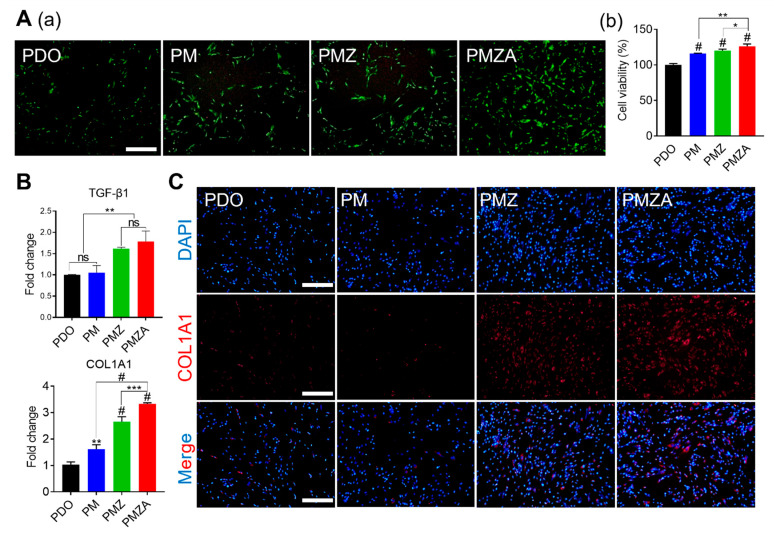
Biocompatibility and collagen deposition ability of the multifunctional PDO threads on hDFs. (**A**) Live/dead staining images (**a**) and cell viability (**b**) at 1 day (Scale bar: 500 μm). (**B**) The mRNA expression levels of collagen deposition-related genes (TGF-b1 and COL1A1). (**C**) Representative ICC images labeled with COL1A1 antibody (Scale bar: 500 μm). * *p* < 0.05, ** *p* < 0.01, *** *p* < 0.001, and ^#^ *p* < 0.0001 indicate statistically significant differences, respectively.

The biocompatibility of multifunctional PDO threads was evaluated by conducting live/dead staining and cell viability assessment using hDFs for 1 day. Overall, excellent biocompatibility was consistently observed across all groups, indicated by the absence of dead cell signals in Figure 2A(a). In addition, the intensity of green fluorescence emitted by live cells exhibited a gradual increase in the sequence of PDO, PM, PMZ, and PMZA. The proliferation of hDFs was enhanced by Mg and Zn ions released from the multifunctional PDO threads. In addition, Mg and Zn ions are known to induce collagen formation [27]. Collagen increases the tensile strength of the skin, so it is effective in improving elasticity and wrinkles [28]. To investigate collagen synthesis by the multifunctional PDO threads, gene expression analysis and immunofluorescence staining were performed. The gene expression of TGF-β1 and COL1A1 was significantly upregulated in the order of the PDO, PM, PMZ, and PMZA (Figure 2B). TGF-β1 as a potent fibrogenic growth factor activates fibroblasts and plays an important role in the wound-healing response [29,30,31]. The COL1A1 gene induces the synthesis of collagen type 1 present abundantly in human skin [32]. Figure 2C displays the representative fluorescence images for COL1A1 expression in hDFs. Higher expression of COL1A1 in hDFs cultured with PMZ and PMZA demonstrated that collagen synthesis was more efficient. Increased proliferation of hDFs and expression of upregulated collagen synthesis-related genes and proteins have shown the potential to improve wrinkles and elasticity by promoting collagen synthesis in the skin tissue [33].

### 3.3. Anti-Inflammatory Effect

Collagen is a vital component for maintaining healthy skin. However, excessive collagen deposition can lead to fibrosis [34]. Numerous approaches have reported successful facial rejuvenation through enhanced collagen synthesis. However, the potential occurrence of fibrosis as a consequence of these treatments has not been thoroughly investigated [35,36,37]. On the other hand, Cooper et al. reported that fibrosis was caused by an inflammatory response in the skin [38]. In particular, the expression of proinflammatory cytokines such as TNF-α, IL-1β, IL-4, IL-6, etc., in fibroblasts is known to induce the overproduction of collagen [39,40].

**Figure 3:**
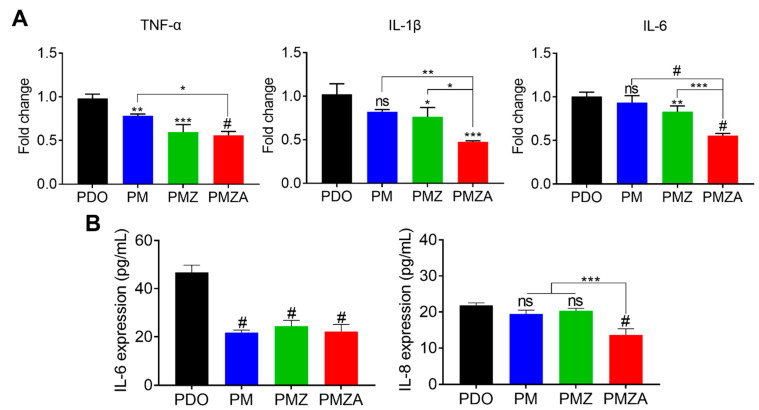
Anti-inflammatory effect of the multifunctional PDO threads. (**A**) mRNA expression levels of inflammation-related genes (TNF-α, IL-1β, and IL-6). (**B**) Protein expression levels of IL-6 and IL-8. * *p* < 0.05, ** *p* < 0.01, *** *p* < 0.001, and ^#^ *p* < 0.0001 indicate statistically significant differences, respectively.

Polyester such as PDO during biodegradation produces acidic byproducts and causes inflammation by acidifying nearby tissue [41]. The expression of inflammatory cytokines was measured to indirectly investigate the occurrence of an inflammatory response and fibrosis during the biodegradation of multifunctional PDO threads. Figure 3A displays proinflammation-related gene expressions of hDFs treated with the by-products of multifunctional PDO threads. Expression levels of TNF-α, IL-1β, and IL-6 gradually decreased in PDO, PM, PMZ, and PMZA due to the pH-neutralizing effect of MH and ZO and the anti-inflammatory effect of ATS [11,42]. Similarly, the quantitative analysis of IL-6 and IL-8 expression as pro-inflammatory cytokines showed a decreasing tendency (Figure 3B). These results suggest that the multifunctional PDO threads can inhibit inflammation and the induction of fibrosis.

### 3.4. Angiogenic Capability

**Figure 4:**
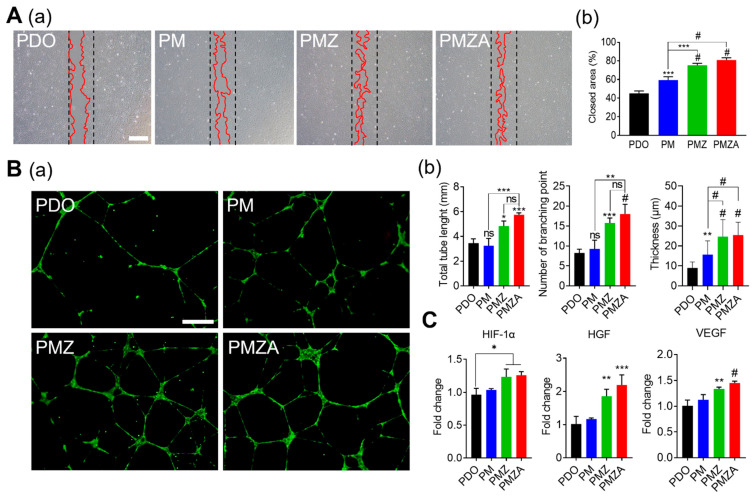
The angiogenic capability of the multifunctional PDO threads on HUVECs. (**A**) Optical images (a) and quantitative closed area (b) of cell migration in wound-healing assay (Scale bar: 500 μm). (**B**) Fluorescence images (a), quantitative total tube length, number of branch points, and tube thickness (b) of the tube-formation assay (Scale bar: 500 μm). (**C**) mRNA expression levels of angiogenic-related genes (HIF-1α, HGF, and VEGF). * *p* < 0.05, ** *p* < 0.01, *** *p* < 0.001, and ^#^ *p* < 0.0001 indicate statistically significant differences, respectively.

Angiogenesis is considered necessary in aesthetic medicine because it inhibits skin aging and induces skin regeneration [43,44,45]. Angiogenesis promotes the formation of neo-tissue by fundamentally supporting the delivery of nutrients and signaling factors and removing waste products [46]. Therefore, the angiogenic capability of multifunctional PDO threads was evaluated for skin regeneration. Figure 4A shows the optical image and quantitative closed area of HUVEC in the presence of an NO donor. The closed area gradually increased in PDO, PM, PMZ, and PMZA. The highest cell migration was exhibited in the groups containing ZA due to ZA reacting with NO donors in the human body to generate NO and the generated NO induced blood vessel formation. A tube formation assay was performed in the presence of the NO donor to demonstrate the pro-angiogenic capability. Figure 4B(a) displays the fluorescence image of the formed tube stained with Calcein AM. The tube formation ability of the PM was comparable to PDO, but PMZ and PMZA showed a significant increase in the total tube length, branch points, and tube thickness. PMZ and PMZA containing ZA reacted with the NO donor to generate NO, and the generated NO greatly assisted the migration and proliferation of HUVEC. The quantitative total tube length (3.45, 3.24, 4.82, and 5.74 mm, respectively), number of branching points (8.25, 9.25, 15.75, and 18, respectively), and thicknesses (8.95, 15.65, 23.84, and 25.42 μm, respectively) are shown in Figure 4B(b). Increased tube length and thickness in PMZ and PMZA indicate the ability to induce angiogenesis by ZA and ATS. qRT-PCR was performed to analyze the gene level affecting cells during angiogenesis. The expression level of NO signaling pathway-related (HIF-1α) and angiogenic-related (HGF and VEGF) genes increased in PMZ and PMZA. The generated NO inhibited the decomposition of HIF-1α by prolyl hydroxylase and led to a hypoxic response despite the presence of oxygen [47]. The hypoxic response induced by NO ultimately increased the expression of angiogenic-related genes. In addition, the ATS released from PMZA was not involved in the hypoxic response but increased the expression of HGF and VEGF.

### 3.5. Histopathological Evaluation

**Figure 5:**
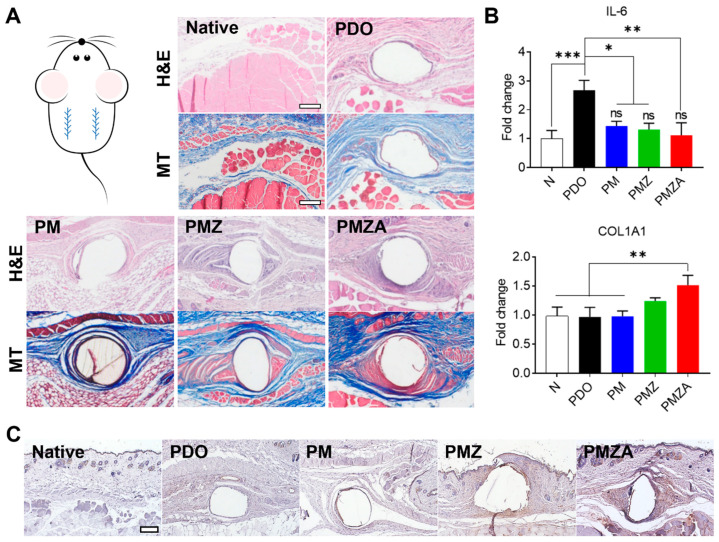
In vivo histological evaluation of the multifunctional PDO threads after 6 weeks of subcutaneous implantation. (**A**) Schematic diagram of implanted PDO threads and H&E and MT staining. (**B**) Relative mRNA expressions related to pro-inflammation (IL-6) and collagen synthesis (COL1A1). (**C**) Immunohistochemistry (IHC) staining images to CD31. * *p* < 0.05, ** *p* < 0.01, and *** *p* < 0.001 indicate statistically significant differences, respectively.

To further explore biocompatibility, the multifunctional PDO threads were subcutaneously implanted for 6 weeks on a BALB/c mouse, as shown in a schematic diagram of Figure 5A. Then, tissues around the multifunctional PDO thread were investigated using the H&E and MT staining analyses (Figure 5A). When a biomaterial is implanted into the tissue, foreign body reactions, such as inflammation and fibrosis, may occur [48]. However, no significant differences in tissue damage or foreign body response were observed in the H&E and MT staining images across all groups. It was quantified using MT staining images to measure the ratio of collagen formed (Figure S4). The proportion of collagen formed meaningfully increased in PMZ and PMZA. As described earlier, fibrosis results from excessive collagen formation triggered by an inflammatory response. To investigate fibrosis occurrence, the expression of genes related to inflammation (IL-6) and collagen synthesis (COL1A1) was measured (Figure 5B). The PM, PMZ, and PMZA groups exhibited IL-6 expression levels similar to the native group. In contrast, the PDO group showed increased IL-6 expression, likely due to acidic by-products. Moreover, COL1A1 expression was upregulated in the PMZ and PMZA groups. These results reveal that non-inflammatory collagen synthesis, rather than fibrosis, was induced in the PMZ and PMZA groups. To investigate the formation of new blood vessels, immunohistochemistry analysis was performed to identify the expression of CD31 as an endothelial marker in Figure 5C. The expression of CD31 was not significantly observed in PDO and PM. On the other hand, the expression of CD31 in PMZ and PMZA was highly expressed in the periphery of transplanted threads. The blood vessels created around the implanted thread can play a major role in regenerating the skin as it can help supply nutrients and remove waste to and from surrounding tissues smoothly.

## 4. Conclusions

PDO thread coated with surface-modified inorganic particles (MO and ZA) and ATS capable of NO generation and sustained drug release was successfully developed using ultrasonic coating technology. This multifunctional PDO thread exhibited excellent biocompatibility, as evidenced by increased cell viability. It demonstrated the ability to regenerate skin through collagen synthesis and anti-inflammatory and angiogenic effects. Histopathological evaluation using a mouse model indicated that the multifunctional PDO threads did not induce significant tissue damage and foreign body response. Also, they stimulated non-inflammatory collagen synthesis in the surrounding tissues, not fibrosis. These results may improve skin elasticity, reduce wrinkles, and improve overall skin regeneration without inducing fibrosis or adverse reactions. This research suggests the potential for innovative biocompatible materials with multifunctional properties to advance cosmetic and regenerative medicine.

## Figures and Tables

**Table 1:** The loading amount of MH, ZO, and ATS on the multifunctional PDO threads.

Sample	Loading Amount (μg/cm)		
	MH	ZO	ATS
PDO	-	-	-
PM	24.49	-	-
PMZ	22.04	10.14	
PMZA	22.93	13.73	10.19

## Data Availability

The data presented in this study are available upon request from the corresponding author.

## Supplementary Materials

The following supporting information can be downloaded at: https://www.mdpi.com/article/10.3390/pharmaceutics15092220/s1, Figure S1: Characterization of surface-modified MH (MO). (A) ATR-FTIR spectra, (B) TGA thermograms, and (C) size distribution; Figure S2: Characterization of surface-modified ZA. (A) ATR-FTIR spectra, (B) TGA thermograms, and (C) size distribution; Figure S3: Mechanical properties of the multifunctional PDO threads. (A) Tensile strength, (B) elongation, and (C) Young’s modulus; Figure S4: Quantitative collagen volume fraction using Masson’s staining images.
