# Supplementary material for: Multifunctional PDO Thread Coated with Mg(OH)2/ZnO Nanoparticles and Asiaticoside for Improved Facial Lifting
Source: Pharmaceutics. 2023 Aug 28;15(9):2220. doi: 10.3390/pharmaceutics15092220 (PMC10535954; doi:10.3390/pharmaceutics15092220)
Supplement: Supplementary file 1 [file pharmaceutics-15-02220-s001.zip › pharmaceutics-2552471-supplementary.pdf]

Multifunctional PDO Thread Coated with $Mg(OH)_2/ZnO$ Nanoparticles and Asiaticoside for Improved Facial Lifting

Dong Min Kim[1,†], Seung-Woon Baek[1,2,3,†], Jeong Min Park[1,†], Da-Seul Kim[1], Semi Lee[1], Jun-kyu Lee[1], Chun Gwon Park[2,3] and Dong Keun Han[1,*]

[1]Department of Biomedical Science, CHA University, 335 Pangyo-ro, Bundang-gu, Seongnam-si, Gyenggi 13488, Korea

[2]Department of Biomedical Engineering, SKKU Institute for Convergence, Sungkyunkwan University (SKKU), 2066 Seobu-ro, Jangan-gu, Suwon-si, Gyenggi 16419, Korea

[3]Department of Intelligent Precision Healthcare Convergence, SKKU Institute for Convergence, Sungkyunkwan University, 2066 Seobu-ro, Jangan-gu, Suwon-si, Gyenggi 16419, Korea

2023. 08. 22

Submitted to Pharmaceutics

*Correspondence: dkhan@cha.ac.kr

†These authors contributed equally to this work.

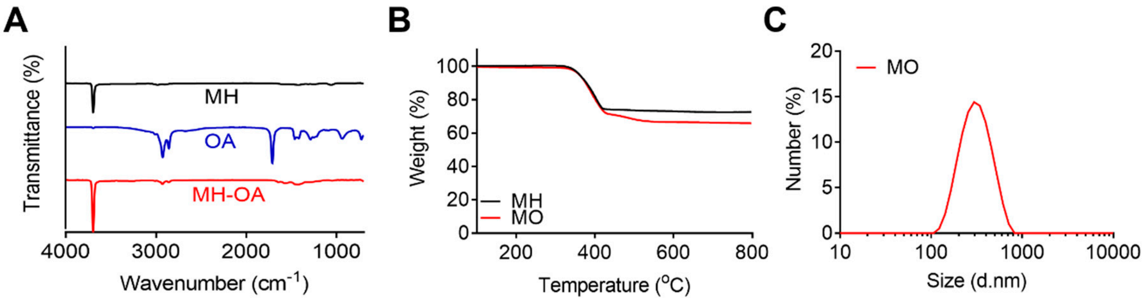

Figure S1. Characterization of surface-modified MH (MO). (A) ATR-FTIR spectra, (B) TGA thermograms, and (C) size distribution.

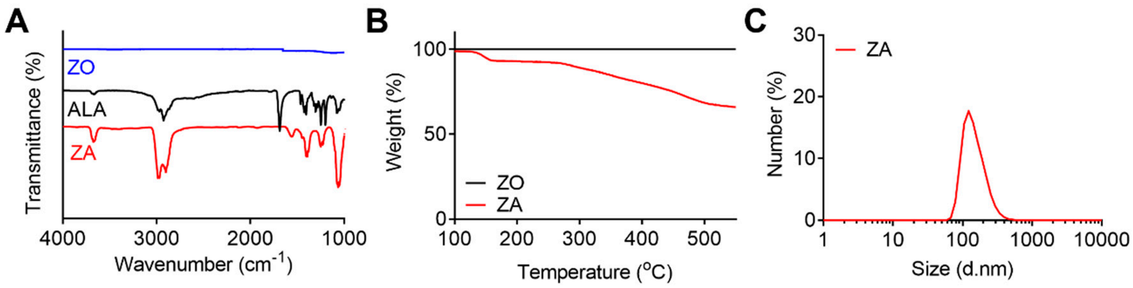

Figure S2. Characterization of surface-modified ZO (ZA). (A) ATR-FTIR spectra, (B) TGA thermograms, and (C) size distribution.

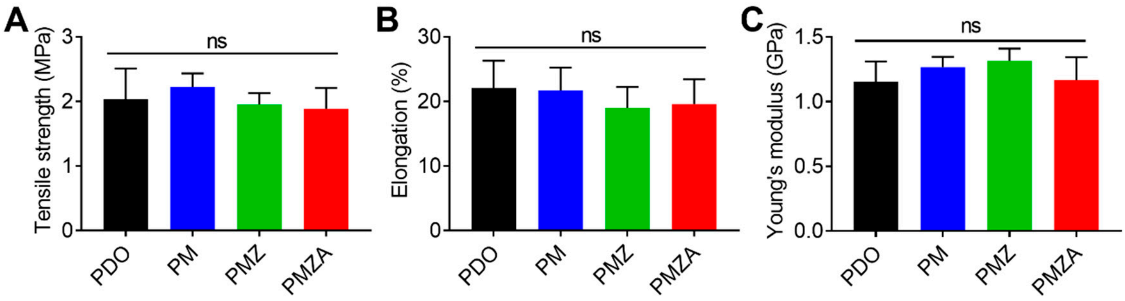

Figure S3. Mechanical properties of the multifunctional PDO threads. (A) Tensile strength, (B) elongation, and (C) Young's modulus.

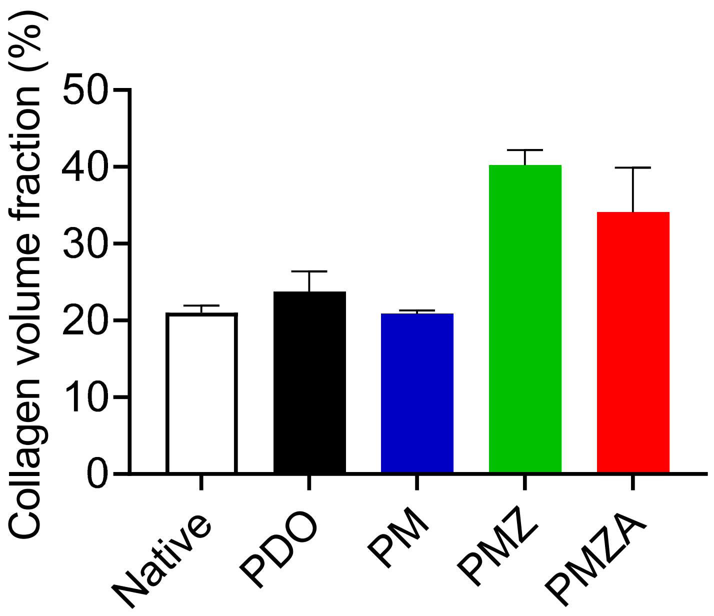

Figure S4. Quantitative collagen volume fraction using Masson's staining images.
